# Organic Matter and Water Addition Enhance Soil Respiration in an Arid Region

**DOI:** 10.1371/journal.pone.0077659

**Published:** 2013-10-18

**Authors:** Liming Lai, Jianjian Wang, Yuan Tian, Xuechun Zhao, Lianhe Jiang, Xi Chen, Yong Gao, Shaoming Wang, Yuanrun Zheng

**Affiliations:** 1 Key Laboratory of Resource Plants, Beijing Botanical garden, West China Subalpine Botanical Garden, Institute of Botany, Chinese Academy of Sciences, Beijing, China; 2 University of Chinese Academy of Sciences, Beijing, China; 3 Xinjiang Institute of Ecology and Geography, Chinese Academy of Sciences, Urumqi, Xinjiang, China; 4 Inner Mongolia Agricultural University, Hohhot, Inner Mongolia, China; 5 Key Laboratory of Oasis Ecological Agriculture of Xinjiang Bingtuan, Shihezi, Xinjiang, China; University of California, Merced, United States of America

## Abstract

Climate change is generally predicted to increase net primary production, which could lead to additional C input to soil. In arid central Asia, precipitation has increased and is predicted to increase further. To assess the combined effects of these changes on soil CO_2_ efflux in arid land, a two factorial manipulation experiment in the shrubland of an arid region in northwest China was conducted. The experiment used a nested design with fresh organic matter and water as the two controlled parameters. It was found that both fresh organic matter and water enhanced soil respiration, and there was a synergistic effect of these two treatments on soil respiration increase. Water addition not only enhanced soil C emission, but also regulated soil C sequestration by fresh organic matter addition. The results indicated that the soil CO_2_ flux of the shrubland is likely to increase with climate change, and precipitation played a dominant role in regulating soil C balance in the shrubland of an arid region.

## Introduction

Soil respiration (R_S_) is considered the second largest terrestrial carbon (C) flux [1]. This large flux is estimated at ∼98±12 Pg/yr, which is an order of magnitude larger than fossil fuel combustion [2], and atmospheric concentration of CO_2_ has increased at 1.9 ppm/yr during the last 10 years [3]. Therefore, any alterations in soil CO_2_ efflux could potentially exacerbate greenhouse gas induced climate warming [4]. The response of R_S_ to climate change is a critical component in predicting the possible changes in the global C cycle and the climate feedbacks [5]. Generally, elevated CO_2_ is assumed to stimulate primary production and C input into soil [6], and additional soil C sequestration could mitigate climate change [7]. As the decomposition of soil organic matter is a temperature dependent process, global warming can also increase soil CO_2_ flux to the atmosphere [5]. Thus, there could be positive or negative feebacks between the elevated CO_2_ and soil C sequestration, and how R_S_ changes under elevated CO_2_ will determine whether the additional C input will be sequestered in soil. Although many simulation experiments [8], [9] and modelling analyses [10] have suggested that R_S_ should change with climate, the responses of R_S_ are not well understood [2], [11].

Soil temperature, moisture and substrate concentration have long been identified as the main abiotic controlling factors of R_S_ [4], [12] and are used as the fundamental parameters in R_S_ simulating models [4], [13]. Temperature can affect almost all biochemical and physiological aspects of the respiration process [14]. The R_S_-temperature relationship has been suggested to be a critical component in predicting global C cycle feedbacks to climate warming [4], [5]. The temperature sensitivity of R_S_ is often described by Q_10_, the factor by which R_S_ changes with a 10°C rise in temperature. The global mean Q_10_ value was reported at ∼1.5 [2], [15]. Despite the many studies across different temporal and spatial patterns on this subject [11], [16], some aspects remain unclear due to the complexity of the R_S_ process and the effects of environmental factors [12], [17]. One important question is the effects of soil temperature with depth on Q_10_ value estimation, because of the phase shift of temperature fluctuations with soil depth increasing [17], [18]. Therefore, the temperature measurements at different soil depths should be considered in field experiments.

Soil moisture plays a crucial role in microbial growth and activity [19]. The activities of the microbes need a certain range of soil water content, and will be depressed as the soils continue to dry [8]. At low soil water potentials, the contact with available substrate and physiological performance of microbes are limited [20]. All these could inhibit the biochemical processes underground and so lower the R_S_ [19]. According to the Fourth Assessment Report of IPCC, significantly increased precipitation has been observed in central Asia [3]. The changed precipitation is anticipated to have great effects on plant growth, plant physiology and ecosystem productivity, and will influence R_S_ dynamics [7], [21].

Under elevated atmospheric CO_2_ concentrations, the stimulated plant productivity can supply more substrate for soil microbes. It has been shown that addition of a small amount of substrate (e.g. cellulose) to soil can strongly increase the total soil respiration and remain constant after the additional substrate exhaustion, which would lead to a soil C loss [22]. However, some studies suggest that increased C inputs could enhance soil C sequestration in terrestrial ecosystems [23], [24]. Therefore, it is necessary to clarify the balance between the organic matter input to the soil and the R_S_ flux for a better understanding of C cycling under climate change.

Arid regions occupy ∼20% of the global terrestrial surface [25], and the amount of soil C stored in arid ecosystems is huge [26]. Although much research has been conducted to investigate the climate change soil C cycle, arid regions have received relatively less attention [27]. Arid ecosystems have been predicted to be one of the most responsive ecosystem types to global climate change [28]. Further, the significant potential C sink capacity in soils of arid ecosystems through restorative management makes arid regions more important for the sequestration of soil C in view of global climate change [26].

The Xinjiang Uygar Autonomous Region (XUAR) in northwest China covers over one sixth of China's land area and includes the majority of the country's arid areas [29]. Widely distributed saline/alkaline soils and low precipitation are two principal characteristics of this region [30]. During the past 50 years, the precipitation in XUAR has significantly increased [31], and this increasing trend is predicted to continue in the future [3]. According to an analysis on climate change of XUAR by Xu and Wei [32], the precipitation in the region had increased by 12.8%–28.8% since 1987. To the soil C pool, the climate changes might bring two contradictory processes: soils may receive more plant C input that will result in a net C sequestration; meanwhile, increasing soil organic C decomposition activities will also stimulate C output. As the CO_2_ flux from R_S_ represents the major pathway for C exchange between the soil C pool and the atmosphere, identifying the response of R_S_ to the impacts of more plant C input and precipitation would help gain a better understanding of C cycling in arid regions under global climate change. Many studies have reported the direct effects of C input [24], [33] and precipitation [27] on C cycling, but little is known about how R_S_ will be affected by the predicted changes in increased C input, precipitation and their interactions, especially in arid regions. An experiment consisting of fresh organic matter and water addition treatments in a shrubland in XUAR was conducted to answer following questions: (1) will the soil have a net C gain or loss from C addition and water addition? (2) how will the relationships between R_S_ and environmental factors change under C addition and water addition? (3) which depth is most suitable for simulating the temperature sensitivity of R_S_ in arid regions?

## Materials and Methods

### Ethics Statement

All necessary permits were obtained for the field studies described. The study sites are managed by the Fukang Station of Desert Ecology, Xinjiang Institute of Ecology and Geography, Chinese Academy of Sciences.

### Study site description

**Figure 1:**
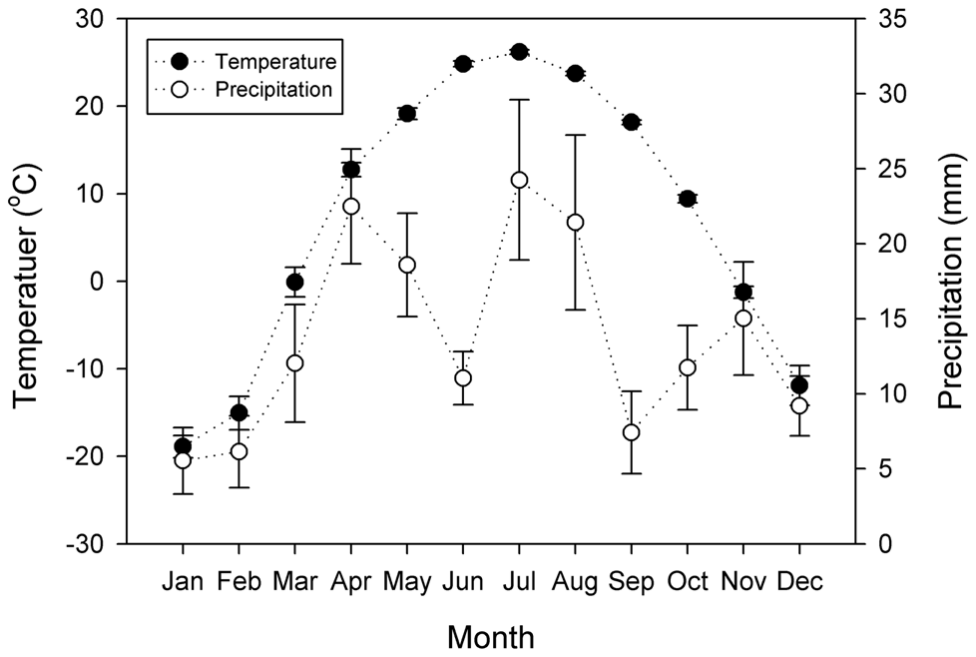
Mean values of temperature and precipitation of the study area (2004∼2011). Vertical bars indicate standard errors of means (*n* = 8).

The study was conducted at the Chinese Academy of Sciences' Fukang Station of Desert Ecology (87° 56′ E, 44° 17′ N, elevation 461 m), an arid area located in the hinterland of northwest China. There is no grazing or other human disturbance existed in the study site. Mean annual temperature is 6.6°C with mean annual rainfall of 160 mm (Figure 1). The soil is clay-loam with high pH (9.38±0.03) and electrical conductivity (1.54±0.36 dS/m). The field site is *Reaumuria soongorica* dominated shrubland, which is a popular native vegetation species in the XUAR.

### Experimental design

The experiment used a nested design with fresh added organic matter (FOM) and water (P) as the two controlled parameters. In early June 2011, 27 randomly distributed study plots (3×3 m) were established in a *R. soongorica* community with uniformly distributed vegetation. Adjacent plots were at least 2 m apart for mitigating buffering effects.

It was difficult to add FOM to the soil surface without being disturbed by the wind in spring. Soil layers deeper than 20 cm can only receive extremely small amounts of plant C and the SOC is relatively stable (Fontaine et al., 2007). Approximately 76% of feeder roots of the *R. soongorica* are located above 20 cm [30]. Therefore, the 20–30 cm layer was chosen as the FOM input layers.

FOM was added to the 20–30 cm deep soil to increase the SOC by 0% (F0, no FOM input), 5% (F1, 33.64 g/m^2^), and 10% (F2, 67.28 g/m^2^). Based on the long-term precipitation data from the nearby weather station of Fukang research station, three water addition treatments were applied weekly at rates equivalent to 0% (P0, no additional irrigation), 50% (P1, approximately 2.43 mm), and 100% (P2, approximately 4.87 mm) increased precipitation. Two water addition regimes (50% and 100% of normal weekly precipitation) used in this study was set as about two and four times of average precipitation increase from 1987 to 2001 [32] in order to get much more significant effect of water addition for well understanding the potential results of water increase. At each water addition plot, water was added with the sprinkling can manually. A randomized block design was used for the plots, with the three FOM treatments nested within the three water addition treatments with three replications.

At the end of the growing season in 2010, plant litter was collected from an adjacent *R. soongorica* community site as the FOM for the experiment. After collection, impurities such as lumps of soil were removed and the plant material was dried at 65°C in an oven for 48 h, and then stored at 4°C until use. Before use in the field experiments, the plant materials were ground and sieved to 2 mm. The C, N content and the C: N of the FOM were 44.42±3.31% (mean ± SE, n = 5), 3.18±0.08% (n = 5) and 13.94±0.97 (n = 5), respectively.

To prepare the plots, dead plant tissues and litter from the soil surface was collected (a very small amount), and replaced after the initial plot treatments had been completed. Next, the top 0–20 cm soil was carefully removed in layers, taking care to keep the soils in their original shape and structure, and ensuring that the plants and roots were not badly affected. The FOM was then added. Finally, the top soil was replaced in its original position, and the gaps between the soil blocks were filled with soil from the 0–10 cm layers collected from an adjacent area. The F0 plots were treated the same as the added FOM treatment plots.

### Soil CO_2_ flux measurements

Soil respiration was measured with an LI-8100 automated soil CO_2_ flux system equipped with an LI-COR 8100-103 chamber (LI-COR chamber volume of 4843 cm^3^, Lincoln, NE, USA). At each plot, one PVC collar (20.3 cm inner diameter and 15 cm high) was inserted into the soil with 3 cm exposed above the soil surface. Before setting up a PVC collar, litter on the soil surface was cleared. Measurements were conducted every two weeks from June to October. Besides the 27 plots with manual treatments, 5 more PVC collars were set at adjacent natural conditions (NC) as controls, similar to treatment sites and without human disturbances in recent 30 years. Measurements were made from 8:00 to 20:00 in two-hour rounds. To reduce the effects of temperature rise, the plots were separated into two parts and measurements conducted on two consecutive days with sunny conditions.

### Soil temperature and moisture measurements

The soil temperature and soil water content (v/v, %) at 0, 5, 10, 20, 30 and 40 cm depth of each single irrigation controlled sites (F0P0, F0P1 and F0P2) were measured automatically with 5TE soil moisture sensors equipped with the EM50 data-logger (Decagon devices, USA).

### Root biomass measurements

Due to the high spatial variability of the shrub roots, root samples were collected from one large square core (25×25 cm, and 60 cm deep) from each plot (27 plots) in October 2011. The roots were carefully separated from these samples and cleaned in water, retaining only apparently living material based on the color, texture, and shape of the roots. Fine roots were classified by their diameter (<2 mm). Then roots were dried at 70°C to a constant mass and weighed. Roots deeper than 60 cm were excluded because very few roots reach that depth.

### Analysis of soil physical and chemical properties

Soil samples were collected from 0–30 cm and sieved to 2 mm immediately and stored at 4°C for microbial biomass C (MBC) analysis. MBC was determined using the fumigation-extraction method [34]. SOC was measured using the method described by Nelson and Sommers [35]. The pH (1∶5 solid–water ratio) and EC (1∶5 solid–water ratio) were determined with a Eutech PC700 pH/EC meter (Thermo Fisher Scientific Inc., Waltham, Massachusetts, USA).

### Data analysis

The temperature dependence of R_S_ was fitted with an exponential function [36]:
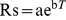
(1)where a and b are fitted constants, and *T* is the soil temperature (°C).

From the equation 1, the Q_10_ was calculated as:
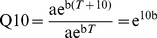
(2)Where b is the fitted constant in equation 1.

A linear function was used to describe the relationship between R_S_ and soil moisture [37]:
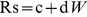
(3)where c and d are fitted constants, and *W* is the soil moisture.

An exponential-exponential function was used to describe effects of soil temperature and soil moisture on Rs [38]:
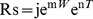
(4)where j, m and n are fitted constants, *W* is the soil moisture, and *T* is the soil temperature (°C).

The model residuals or residual sum of squares (RSS) were used to evaluate the model performance.

To investigate the R_S_ changes induced by a single treatment factor, changes in R_S_ were calculated between F0 and F1, F0 and F2 under same water treatment; and R_S_ changes between P0 and P1, P0 and P2 under same FOM treatment. The changes were calculated as:

FOM (or Water) induced change percentages in 
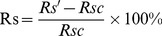
(5) for FOM induced change percentages in R_S_, the *R_S_′* is soil respiration with F1 or F2 treatment, *R_S_c* is soil respiration with F0 treatment, under the same water condition; for water induced changes percentages in R_S_, *R_S_′* is soil respiration in plots with P1 or P2 treatment, *R_S_c* is soil respiration in plots with P0 treatment, under same FOM treatment condition.

All statistical linear and nonlinear regression analyses, multiple comparisons including one-way ANOVA and homogeneity of variance tests were performed with SPSS 13.0 software [39]. Multiple comparisons of means for different treatments were analyzed using Tukey's test.

## Results

### Effects of measurement depth on temperature sensitivity of soil respiration

**Figure 2:**
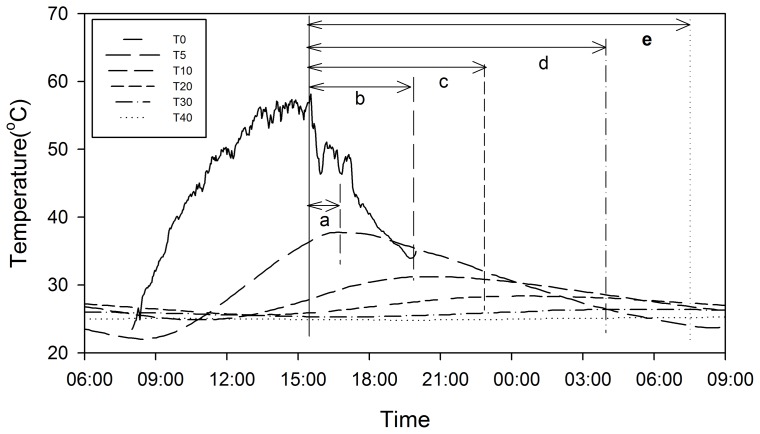
Daily courses of soil temperature at different measuring depths at the adjacent natural conditions (data of Jun-21 and 22). T0, T5, T10, T20, T30 and T40 represent temperature measured at 0, 5, 10, 20, 30 and 40-delay of daily highest temperature from T0, and values are 60, 240, 420, 740 and 960 sec minutes for a, b, c, d and e, respectively.

**Figure 3:**
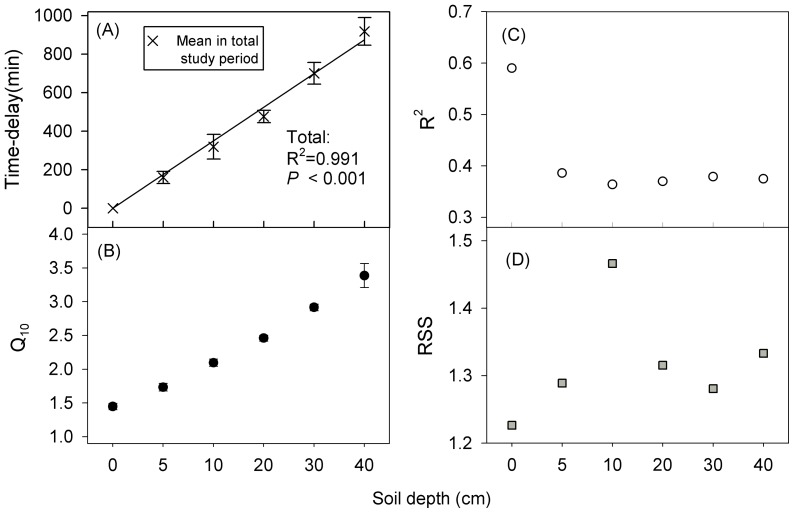
Dependence of the time-delay of daily highest temperature from T0 (mean ± SE), values of Q_10_, R^2^ and RSS on the depth of soil temperature measuring point. Sub-Fig (A), (B), (C) and (D) represent time-delay from T0, values of Q_10_, R^2^ and RSS, respectively. Q_10_: the temperature sensitivity of R_S_; RSS: residual sum of squares of the exponential function.

R_S_ ranged from 0.02 to 0.85 µmol CO_2_ m^–2^ s^–1^ with an average of 0.47±0.02 µmol CO_2_ m^–2^ s^–1^ for the NC plots. The temperature varied from 2.77–60.16, 9.42–38.00, 13.04–32.64, 15.29–29.35, 16.64–28.87 and 17.55–28.11°C for the 0, 5, 10, 20, 30 and 40 cm soil layers, respectively. An increasing time delay of the daily highest value of soil temperature and lower amplitudes of daily temperature with deeper soil depths was observed (e.g. temperature of Jun-21 and 22, Figure 2). The time delay of the temperature between 0 cm and other soil depths increased significantly with a linear relationship in the whole measuring period (*P*<0.001, Figure 3A).

Q_10_ calculated from seasonal values of R_S_ and temperature increased significantly with depth of the soil temperature measuring point, from 1.45±0.05 (at 0 cm) to 3.39±0.18 (at 40 cm) (Figure 3B). The R^2^ and RSS (residual sum of squares) of the exponential relationship between R_S_ and temperature showed a better simulating effect at the 0 cm temperature, because the highest R^2^ and lowest RSS were observed (Figure 3C, D). For reducing the uncertainty in simulating the relationship of R_S_ and temperature, due to time delay of the temperature at deeper soil layers, only 0 cm temperature was used for Q_10_ calculations. Further, the equations derived from 0 cm temperature had higher R^2^ and lower RSS compared with that derived from temperature at other soil layers.

### Temperature and soil moisture

**Figure 4:**
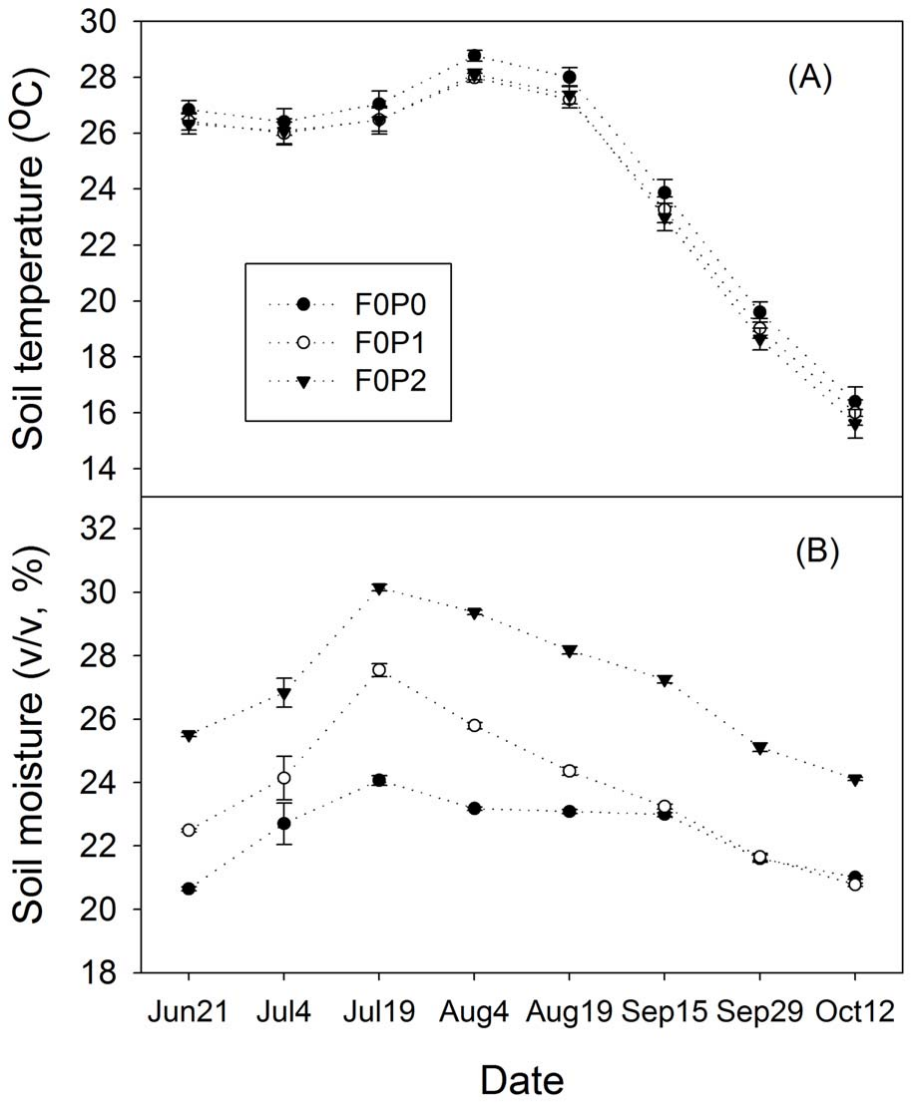
Mean values of temperature and soil moisture under different water addition regimes without FOM treatments. Sub-Fig (A) and (B) represent temperature and soil moisture, respectively. Vertical bars indicate standard errors of means (*n* = 3). FOM: fresh organic matter; F0: no FOM input; P0, P1, P2: no added water, 50% and 100% increase in water addition, respectively.

During the study period (June–October), soil temperature at 0–30 cm had no significant differences under the three precipitation regimes, although slightly lower temperatures were observed in the plots with added water (Figure 4A). Water addition significantly increased soil moisture at 0–30 cm (*P*<0.001).

In contrast to the P0 plots, the relative increase in mean soil moisture values at P2 were 3.1–6.2% (v/v) through the whole study period, and the relative increase in mean soil moisture at P1 plot was 1.3–3.5% (v/v) from Jun 21 to Aug 19, but there were no significant differences between the soil moisture of P0 and P1 treatments during the last two months (Figure 4B).

### Dynamics of soil respiration in the study period

**Figure 5:**
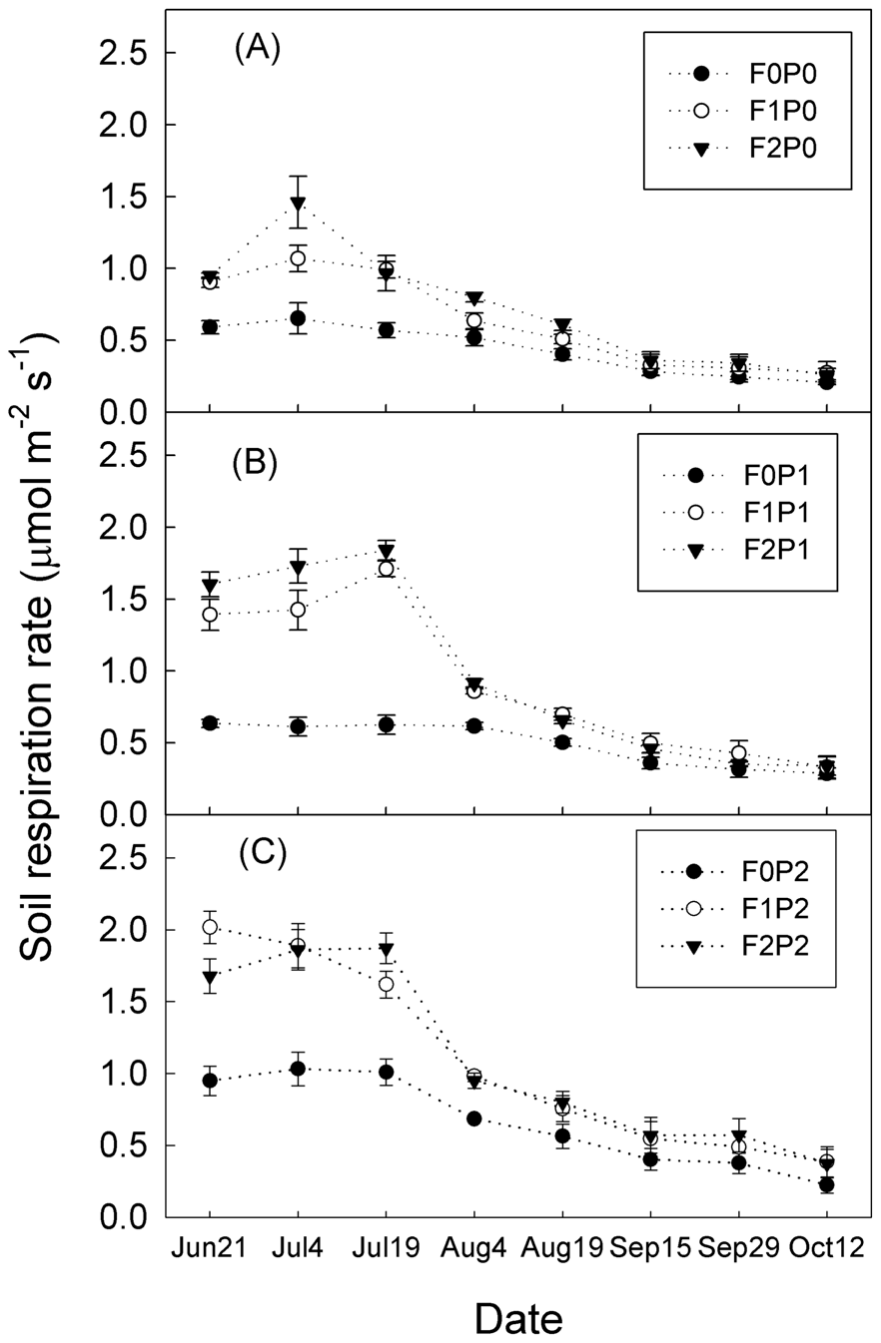
Daily means of soil respiration (mean ± SE) under different treatments. Sub-Fig (A), (B) and (C) represent soil respiration rate under P0, P1 and P2 treatments, respectively. Vertical bars indicate standard errors of means (*n* = 3). F1 and F2: 5% and 10% increase in soil organic carbon. Other abbreviations are same as Figure 4.

R_S_ in all treatments showed a seasonal pattern with a single peak curve that occurred in July and began to decrease in August, partly due to the high temperature and lower soil moisture (Figure 4, Figure 5). Under single water addition, the R_S_ variations in F0P0, F0P1 and F0P2 plots were lower than that under FOM addition.

**Figure 6:**
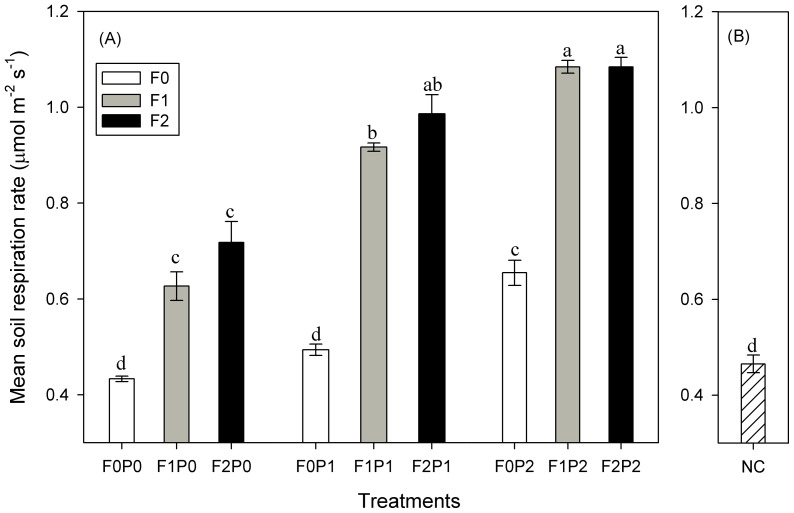
Mean values (mean ± SE) of soil respiration under different treatments and at the natural condition site in total study period. Sub-Fig (A) and (B) represent soil respiration under different treatments and natural site, respectively. Vertical bars indicate standard errors of means (*n* = 3). Bars with different lowercase letters are significantly different from each other at *p*<0.05. Abbreviations are same as Figure 5.

One-way ANOVA showed that there were significant differences among mean R_S_ values of the nine treatments (*P*<0.05; Figure 6A). They were highest at F2P2 and lowest at F0P0 plots, with values of 1.08±0.02 and 0.47±0.02 μmol m^−2^ s^−1^, respectively.

No significant difference was found (*P*>0.05, Figure 6) for the mean R_S_ between the F0P0 (with manual disturbances) treatment and the adjacent natural condition site.

### Effects of FOM addition on soil respiration

**Figure 7:**
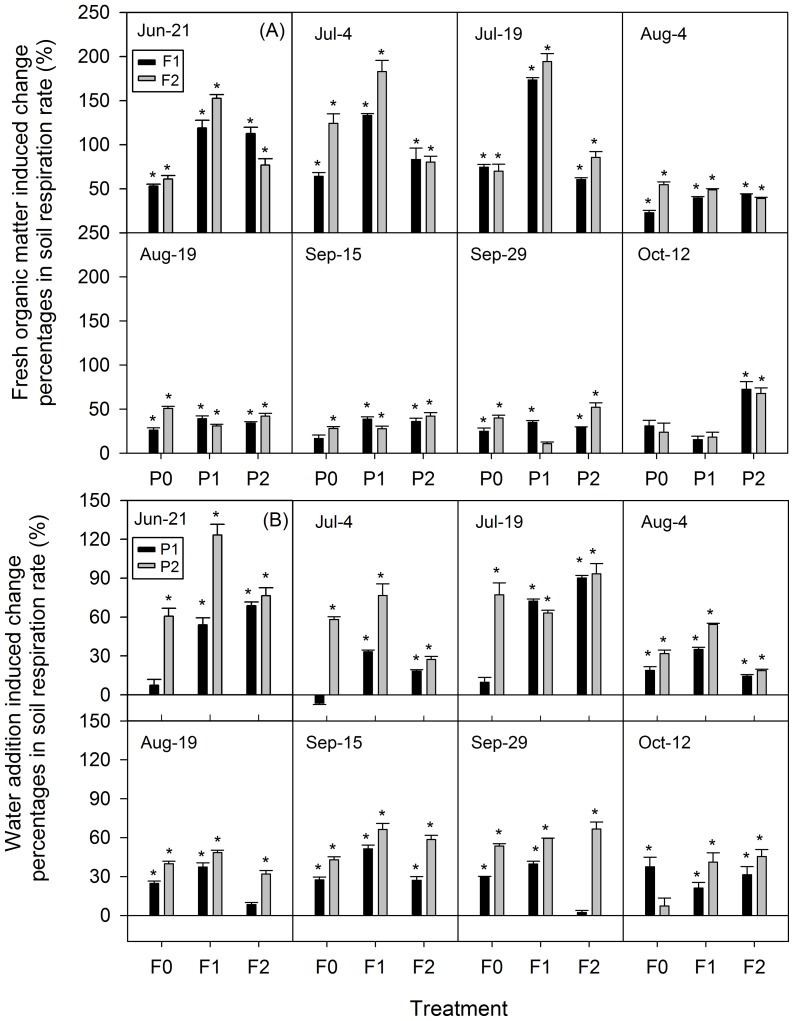
Fresh organic matter and water addition induced change percentages in soil respiration rate (mean ± SE). Sub-Fig (A) and (B) represent fresh organic matter and water addition induced change percentages, respectively. Vertical bars indicate standard errors of means (*n* = 3). * represent significant different compared with controls at *P*<0.01. Other abbreviations are same as Figure 4.

The FOM addition to soil significantly increased mean R_S_ of the total study period (Table 1, Figure 6A). Compared with R_S_ in plots without FOM addition for each precipitation treatment (F0P0, F0P1 and F0P2), the greatest increases induced by FOM addition were observed in the first two months of the experiment (Jun-21 to Jul-19), and varied from 52 to 194% (P<0.01, Figure 7A). The FOM addition-induced increase in R_S_ was larger than that induced by water addition at this period, and then the stimulating effects on R_S_ declined from Aug-4 to Oct-12 (Figure 7).

**Table 1:** Results (*F*-Values) of ANOVA with fresh organic matter (FOM) and water addition, and their interactions on soil respiration.

Source	df	F
FOM (F)	2	188.703***
Water addition (P)	2	99.136***
F×P	4	12.571***

*** , *P*<0.001.

### Effects of water addition on soil respiration

The water addition had a significant effect on variations of R_S_ (Table 1). During the whole study period, P1 and P2 treatments stimulated mean R_S_ by 14 and 51%, 46 and 73% and 37 and 51% under the F0, F1 and F2 treatments, respectively (Figure 6A). R_S_ changes induced by water addition were also higher during the first two months (except some values on Jul-4). Water addition induced higher increases in R_S_ on plots with FOM treatment (F1 and F2) than those without FOM treatment (F0) on most of measuring days (Figure 7B).

### Variation in soil respiration related to temperature, soil moisture, microbial biomass and fine root biomass

**Figure 8:**
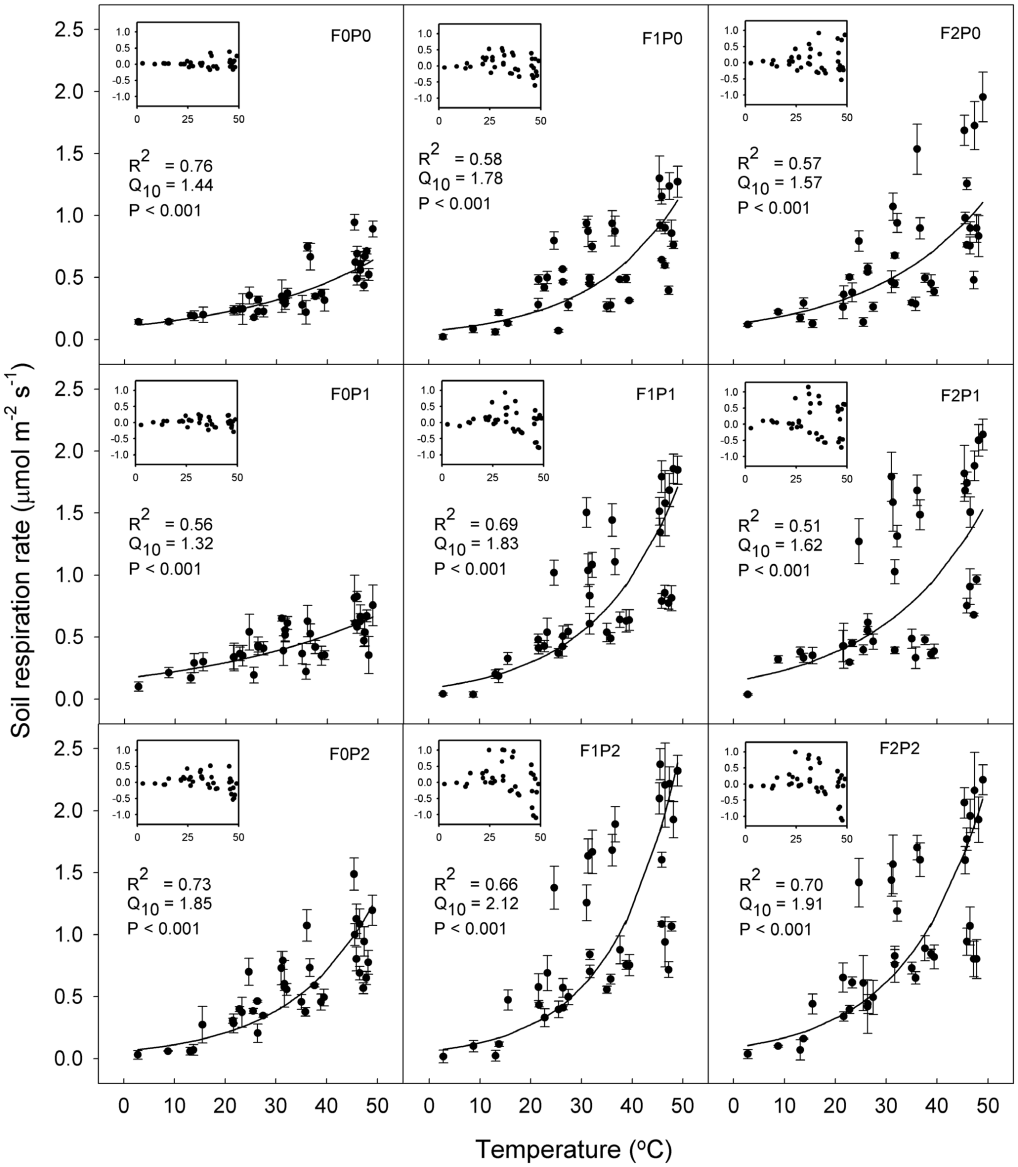
Relationship between soil respiration (mean ± SE) and soil temperature under different FOM and water addition treatments. An exponential was used to describe the relationship. Vertical bars indicate standard errors of means (*n* = 3). Inserts represent the residuals from the equation. Other abbreviations are same as Figure 4.

Due to the depression of the mesophilic microbial community by the high temperature, relationship between R_S_ and the temperature was calculated used data below 50°C. The variations in R_S_ showed a significant exponential relationship with temperature for all treatments (P<0.001), which explained 51–76% of the variation in R_S_ (Figure 8). The residual distributions in all treatments indicated a high simulation of R_S_ at a relatively lower temperature range (∼<30°C). Q_10_ for the nine treatments ranged from 1.32 to 2.12. Comparing with the Q_10_ for controls plots (F0P0), FOM and/or precipitation treatments increased the Q_10_ (except Q_10_ for F0P1).

When soil moisture was taken as a single controlling factor of R_S_, the linear equations explained 29–64% of the R_S_ variation (*P*<0.001, Table 2) and the fitted equations were better in the treatments without water addition (R^2^ = 0.43–0.64).

**Table 2:** Fitted relationships of soil respiration (µmol m^–2^ s^–1^) with soil moisture (at 10 cm soil depth) (*W*, %) in all treatments.

	Functions	R^2^	*P*
F0P0	R_S_ = −0.55+4.18*w*	0.43	***
F1P0	R_S_ = −1.27+8.04*w*	0.58	***
F2P0	R_S_ = −1.81+10.72*w*	0.64	***
F0P1	R_S_ = −2.41+11.62*w*	0.50	***
F1P1	R_S_ = −7.31+32.95*w*	0.45	***
F2P1	R_S_ = −8.63+38.51*w*	0.44	***
F0P2	R_S_ = −5.46+23.99*w*	0.38	***
F1P2	R_S_ = −9.08+39.86*w*	0.29	***
F2P2	R_S_ = −9.25+40.52*w*	0.33	***

F0, F1, F2: no FOM input, 5% and 10% increase in soil organic carbon; P0, P1, P2: no added water, 50% and 100% increase in water addition, respectively. Other abbreviations are same as Table 1.

Soil moisture and temperature together could improve the correlation coefficients of the regression equation for R_S_ (*P*<0.001, *R* ^2^ = 0.61–0.82) in all treatments (Table 3). Similarly, the fitted relationships were better under plots without water addition.

**Table 3:** Fitted relationships of soil respiration (µmol m^–2^ s^–1^) with soil moisture (at 10 soil depth) (*W*, %) and soil temperature (*T*, at soil surface, °C).

	Functions	R^2^	*P*
F0P0	R_S_ = 0.047e^0.300*T*^e^4.407*W*^	0.82	***
F1P0	R_S_ = 0.014e^0.045*T*^e^8.352*W*^	0.65	***
F2P0	R_S_ = 0.016e^0.029*T*^e^10.897*W*^	0.76	***
F0P1	R_S_ = 8.05E^−03^e^0.020*T*^e^13.332*W*^	0.61	***
F1P1	R_S_ = 2.32E^−03^e^0.051*T*^e^15.926*W*^	0.72	***
F2P1	R_S_ = 1.59E^−05^e^0.025*T*^e^39.975*W*^	0.66	***
F0P2	R_S_ = 5.27E^−03^e^0.056*T*^e^10.273*W*^	0.74	***
F1P2	R_S_ = 0.013e^0.072*T*^e^6.275*W*^	0.66	***
F2P2	R_S_ = 8.12E^−03^e^0.06*T*^e^10.035*W*^	0.71	***

Other abbreviations are same as Table 1.

When soil temperature, soil moisture, microbial biomass (MB) and fine root biomass (FRB) were used, the regression equation, R_S_ = 0.501T +0.023 W +0.033FRB –0.002 MB –16.36 (*P* = 0.018, *R* ^2^ = 0.92), could be built and *R* ^2^ was improved. When stepwise regression was used to build an equation between R_S_ and the four factors, only root biomass significantly influenced Rs (*P*<0.001, *R* ^2^ = 0.86).

## Discussion

### Measurement depth and soil respiration

Poor understanding of the temperature sensitivity of R_S_ results in uncertainty in climate models [11], [16]. In many studies, Q_10_ was calculated without accounting for the depth of the soil temperature measuring point [40]. For example, studies have measurements at 2 cm depth [17], 5 cm depth [38] and 10 cm depth [41]. Because heat conductance from the soil surface to below-ground needs a period of time, a time-lag exists between the temperature at the soil surface and other layers. Previous studies [18], [37] stated that Q_10_ values generally increased with increasing soil depth, because of the lower temperature fluctuations in deeper soil layers. Similar results were also found in this study. Besides that, the highest R^2^ and lowest RSS of the exponential relationship between R_S_ and temperature at the soil surface were found (Figure 3). Therefore, 0 cm (soil surface) was considered as the appropriate measuring point for field experiments in arid areas, which was also reported by Pavelka et al. [42].

### FOM addition and soil respiration

Elevated atmospheric CO_2_ concentration can enhance plant growth [6], [43], and the enhanced plant productivity can increase soil C input. The importance of C substrate to R_S_ has been well documented [12], [44]. In the shrubland, FOM addition increased both daily (Figure 5) and the total study period (Figure 6A) R_S_ averages. A similar stimulation of R_S_ by increased organic matter input to soil was also reported by Sulzman et al. [45]. One reason for this variability is that enhanced FOM input supplied more easily decomposed substrate for the soil microbes which would stimulate both the microbial activities and biomass [22]. Besides that, nutrients released from the FOM decomposition can enhance root growth [46], thus increasing CO_2_ from root respiration.

During the study period, higher R_S_-stimulating effects by FOM addition were observed in the first two months (Figure 7A). This can be attributed to the “priming effect” [47], which means that fresh organic C can supply energy and nutrients for soil microbes, and can therefore, accelerate soil organic C (SOC) mineralization [48]. Thereafter, although the FOM-induced change in R_S_ declined, it remained positive and constant till the end of the experiment (Fig 6A). This might be due to the fact that the FOM was exhausted during the first two months, and the “priming effect” was inhibited. Previous studies also reported similar results [22], [44]. The results indicated that FOM addition can cause a sustained R_S_ increase, at least throughout this study period from June to October.

In the long-term incubation ranged from weeks to months, the priming effect can be considered as real priming effect (soil organic matter decomposition) if the amount of the primed C is higher than both microbial biomass and the added C [49]. In this study, the initial microbial biomass C was 20.58 g/m^2^ in the 20–30 cm soil layer. Thus, the real priming effect was happened under the F1P1 and F1P2 treatments (Table 4), while under the other FOM addition treatments could not exclude the apparent priming effect.

**Table 4:** Soil C input–emission balanced with different FOM and water addition treatments during study period. Data are in contrast to the controls (F0P0).

Treatments	C added (g/m^2^)	Additional C lost as CO_2_ (g/m^2^)	Soil C input–emission balance (g/m^2^)
F1P0	21.03	11.43±1.77	9.60±1.77
F2P0	42.05	16.83±2.56	25.22±2.56
F0P1	0	3.58±0.69	−3.58±0.69
F1P1	21.03	28.57±0.52	−7.54±0.52
F2P1	42.05	32.69±2.35	9.36±2.35
F0P2	0	13.08±1.56	−13.08±1.56
F1P2	21.03	38.48±0.79	−17.45±0.79
F2P2	42.05	38.47±1.17	3.58±1.17

Other abbreviations are same as Table 1.

C lost as CO_2_ (g/m^2^)  =  mean soil respiration of the whole period (µmol CO_2_ m^–2^ s^–1^) ×12 g × 1E^−6^ × time (s); Additional C lost as CO_2_ (g/m^2^)  =  (C lost as CO_2_ in other treatments) – (C lost as CO_2_ in F0P0); Soil C input–emission balance (g/m^2^)  =  (addition C lost as CO_2_) – (C added).

The amount of FOM added in the F2 treatment was double that of the F1 treatment, however, the FOM-induced increase in R_S_ between these two treatments did not follow this relationship under the same water conditions. This demonstrated that the effect of FOM addition on the primed CO_2_ amount can be varied with the amount of FOM [49].

### Soil water addition and soil respiration

Soil moisture is generally considered to be a crucial limiting factor for growth and activities of roots and microbes [7], [21] and the diffusion of soluble substrates and oxygen [19] thus indirectly affecting R_S_. Consistent with previous reports [21], [46], this study showed that precipitation treatments stimulated R_S_ of the shrubland. However, some field observations found that precipitation treatments (addition or reduction) did not affect R_S_ in a temperate forest [50] and a tropical rain forest [51]. Borken and Matzner [9] attributed these differences to the stock of plant available water in soil, and stated that R_S_ would not be affected by water treatments until the stock of plant available water was significantly changed. Therefore, there should be an optimum water content that can provide the greatest boost to R_S_. Rey et al. [41] reported that there was a soil moisture threshold, and R_S_ flux increased with moisture content below this threshold, and remains steady above the threshold. Some authors stated that the optimum water range was from 30 to 50% [52]. In this study, the R_S_ was stimulated with an increase in soil moisture (Table 2) and soil moisture in all plots ranged from 20.6 to 30.1% (52.8 to 77.2% of the field moisture capacity) during the experimental period (Figure 4B). This indicated that the soil moisture threshold for R_S_ in the shrubland was >30%, and the predicted precipitation increase will stimulate R_S_ and release more CO_2_ into the atmosphere, although we could not estimate the accurate optimum moisture for the arid soil communities. In arid land, the low soil water content is not ideal for plant growth and decomposers, and may suppress the response of temperature on respiration [53], R_S_ was more dependent on soil moisture. After the manipulation of water addition, the activities of plants and microbes will be enhanced after the relief of water stress, which can increase R_S_ variation. Therefore, soil moisture could better explain the seasonal variations of R_S_ in drier treatments (Table 2).

### Interactive effects among temperature, FOM input and water addition

Temperature has long been identified as a crucial controlling factor on R_S_ [4], [14]. Variations of R_S_ are usually highly correlated with temperature in a positive exponential relationship [36]. Hence, it is often assumed that global warming will stimulate soil respiration and lead to a positive feedback loop between R_S_ and atmosphere CO_2_ [2]. In this experiment, the variation of R_S_ also showed an exponential increase relationship with temperature for all treatments (Figure 8). The temperature sensitivity of R_S_-Q_10_ has received research interest due to the concern about variations of R_S_ under global warming, and the global mean Q_10_ value was reported at ∼1.5 [2], [15]. In this study, the Q_10_ for the control was 1.44, which was closed to the global mean Q_10_ value (1.5). However, more water addition stimulated Q_10_ relative to the controls (Figure 8). Similar results that low soil water content can decrease the temperature sensitivity of R_S_ have been reported by others, and the reason was attributed to the limitation in diffusion of solute substrate [5], [8], [37]. Besides water addition, FOM input was also found to have a stimulating effect on Q_10_ in the study (Figure 8). Similar results that additional FOM amplified the stimulated effects of higher temperature, which was ideal for the increasing of root and microbial biomass, have been reported by previous studies [46]. Arid ecosystems have been predicted to be one of the most responsive ecosystem types to global climate change [28]. Similarly, the result indicated that R_S_ in arid land might change to be more sensitive to global warming if the soil received more organic C and precipitation.

As described above, R_S_ is affected interactively by many factors. In these studies, it was found that interactions of soil moisture and temperature better predict variations in R_S_ for all treatments than single factors (Table 3). Similar results have been found in earlier works [37], [54].

Given that both FOM input and water addition showed positive effects on R_S_, their interactive effects were assumed to be larger. In the results, the synergistic effects of these two treatments on R_S_ increases were found in the first two months of the experiment, thus the effect of FOM addition was larger in watered treatments (Figure 7A), and the effect of water addition was larger in FOM addition treatments (Figure 7B). It can be explained that soil in arid regions only receives a small amount of plant litter input because vegetation is sparse and water is limited [26]. The interactive effects of these two treatments could relieve the stress from lack of substrate availability [19] and therefore cause larger R_S_ changes than the single treatment. However, the synergistic effects decreased afterwards, possibly attributed to exhaustion of the FOM, because the FOM input was more easily decomposed than the SOC [48]. Fontaine et al. [22] stated that supply of organic matter increased the populations of microbes, which could survive on SOC after organic matter exhaustion. This could explain the stability of the stimulated effects of FOM at plots without water addition after FOM exhaustion during the last three months (Figure 7A).

Organic C input and precipitation are predicted to increase in arid regions. In contrast to the controls (F0P0), the soil C input–emission balanced was calculated (Table 4). In our prior study about the effects of treatments (FOM and water addition) on the soil organic C pool, it showed that the SOC in soil of F2 treatment plots were slightly higher than in soil of other treatment plots, but the differences were not significant [55]. In this study, the positive C input–emission balance was found in F2 treatment plots under the same water addition regime (Table 4). Besides that, results of the soil C input–emission balance also indicated precipitation increase played a dominant role in determining whether the soil C pool will get a net sequestration from more organic C input. It was found that water addition not only caused a negative C balance at plots without FOM treatment, but also lowered the soil C sequestration that resulted from FOM input. Furthermore, a soil priming effect caused by organic C input was also reported [22], [44], but it only happened under better soil water conditions.

## Conclusions

As organic C input to soil and precipitation are predicted to increase in arid regions, a better understanding of how R_S_ responds to these changes is essential to predict how soil C dynamics may change with global climate change. In the study, it was concluded that the temperature at the soil surface but not other soil depths can better simulate the relationship between R_S_ and temperature. The FOM and water addition treatments stimulated R_S_ and its temperature sensitivity. In the shrubland of an arid region, precipitation, rather than FOM, played a more dominant role on soil C balance. Water addition enhanced soil C emission, and although soil could get net sequestration by FOM addition without precipitation addition, soil sequestration became negative when precipitation increased under F0 and F1 treatments. These results indicated that the soil CO_2_ flux of this shrubland is likely to increase with climate change, and the critical role of precipitation in soil C balance had implications to future studies conducted at the arid region.
